# Health and Genomic Literacy Among Individuals at Risk for Type 2 Diabetes: A Feasibility Pilot Study

**DOI:** 10.1155/nrp/2672725

**Published:** 2026-10-31

**Authors:** Elisa Airikkala, Marja Kaunonen, Anna-Maija Koivisto, Elina Pimiä, Arja Halkoaho

**Affiliations:** ^1^ Faculty of Social Sciences/Health Sciences Unit Tampere University Arvo Ylpön Katu 34 Tampere 33520 Finland uta.fi; ^2^ Social Services and Health Care Tampere University of Applied Sciences Kuntokatu 3 Tampere 33520 Finland tamk.fi; ^3^ Wellbeing Services County of Pirkanmaa Hospital Services Biokatu 10 Tampere 33520 Finland; ^4^ The Finnish Diabetes Association Näsilinnankatu 26 Tampere 33200 Finland; ^5^ Faculty of Medicine and Health Technology Tampere University Arvo Ylpön Katu 34 Tampere 33520 Finland uta.fi

## Abstract

Health literacy requires new competences as healthcare evolves, particularly when genomics is applied to disease prevention. Genomic literacy has been described from the same foundations as health literacy, but it also has its own unique characteristics, including an emphasis on numeracy. The aim of this longitudinal feasibility pilot study was to assess the health and genomic literacy of participants at risk of Type 2 diabetes, to better understand the factors influencing health and genomic literacy, as well as the relationship between these two literacy perspectives. The Everyday Health Information Literacy (EHIL) instrument, the European Health Literacy Survey Questionnaire (HLS‐EU‐Q16), and genomic literacy measures were used with 60 participants at baseline, and 49 participants completed postsurveys. Participants were informed of their personal polygenic risk score (PRS) for Type 2 diabetes before they filled the postsurveys. The data were analyzed using statistical methods. Health literacy did not change significantly during intervention, but genomic literacy, Section A, scores improved at postsurvey. There was no association between health literacy, EHIL, and genomic literacy, Section A, at baseline; however, a correlation was observed at postsurvey. Motivation was also correlated with health literacy, HLS‐EU‐Q16, and genomic literacy, Sections A and C, at postsurvey. In contrast, education, which appeared to influence genomic literacy, Section A, at baseline, no longer had a significant role at postsurvey. Even brief health counseling and genomic risk information can improve genomic literacy. We recommend that PRS results be disclosed in person, combined with lifestyle counseling. Personal counseling allows for the results to be explained according to the participant’s health literacy and understanding.

## 1. Introduction

Health literacy is a remarkable determinant of health, and low health literacy has been associated with poorer health outcomes [1, 2]. However, an awareness of high‐risk results has been seen to increase motivation to prevent Type 2 diabetes, even when health literacy, genomic literacy, or numeracy were low [3].

The hereditary risk of Type 2 diabetes can be assessed using the polygenic risk score (PRS) testing. In Finland, the PRS is not currently used in public healthcare, but it is available through private clinics, for example, in occupational healthcare, as well as through commercial tests. Understanding health literacy and genomic literacy is important when such genomic testing becomes more widely used in clinical practice. Genomic testing requires increased competence also among nurses, as they have self‐evaluated their level of understanding as poor [4]. When introducing new clinical practices, it is important to study their feasibility and develop PRS‐related information and health counseling. This feasibility pilot study provided valuable experience with PRS disclosure, indicating its potential applicability in diabetes risk and prevention consultations in the Finnish context.

According to Sørensen et al. [5], health literacy entails people’s knowledge, motivation, and competences to access, understand, appraise, and apply health information to make judgments and take decisions in healthcare, disease prevention, and health promotion to maintain or improve quality of life. The definition of health literacy by Ratzan and Parker [6] also shows that the capacity to utilize services is seen as part of health literacy. However, as healthcare services evolve over time, health literacy may also change accordingly. Furthermore, the broad definition of health literacy itself can cover changes in society and eras. Healthy People 2030 updated the individual concept of health literacy to include considering the health of others [7].

As health technology advances, health literacy increasingly requires new knowledge and skills. Genomic literacy is one such competence needed in contemporary healthcare. Genomic literacy, also referred to genetic literacy, genome‐related literacy, or genome information literacy, is defined in terms similar to health literacy, which leads to making appropriate health decisions [8, 9]. In this study, we used the concept of genomic literacy to measure participants’ understanding of genomics‐related topics, although genomic literacy as a concept is itself quite broad. However, terms such as *genetic knowledge* or *genetic information* were used when these were employed in the original studies.

Genomic literacy particularly emphasizes a basic knowledge of genes, genomes, and general genomic science [9]; an appreciation of genetic principles [10]; the interpretation of genomic information [8, 11]; the certainty and uncertainty of genetic information [11, 12]; numeracy [13]; knowledge of how applications of genetic technologies are used [11]; and a knowledge of science [9, 14]. Ultimately, decision‐making also takes place in relation to social [10, 12] and societal issues [10–12]. Daly and Kaphingst [15] have defined the concept as “*sufficient knowledge and understanding of genetic information to allow for effective communication and application of learned information and informed decision-making in personal, societal and professional context.*” However, measuring genomic literacy still requires clarification due to its broad concept. Many measures focus on the ability to recall various genetic terms rather than assessing the understanding and interpretation [15]. Although genomic technology has advanced, measures do not usually assess the competence that would be needed when using current genome‐wide tests [16, 17].

Social determinants influence health literacy, and higher education has been associated with higher health literacy [2, 3], genomic literacy [3, 18, 19], and numeracy [3]. Genomic risk comprehension has been associated with the level of education [20], but with age, health literacy [2], genetic knowledge [21, 22], and health numeracy [3] decreased. Younger people have been found to be more likely to recall their exact lifetime risk estimate and their risk‐increasing alleles [23]. However, genomic risk comprehension and risk interpretation have also not been found to be associated with age [20]. The influence of gender is not clear‐cut. While women have been reported to have a greater understanding of the principles of genetic risk factors and better knowledge of how to apply this information than men [24], Chapman et al. [18] unexpectedly found that men scored higher than women on genomic knowledge.

Previous studies have shown that those with limited health literacy also had less genetic knowledge [22]. Those with higher health literacy were significantly more familiar with basic genetic terms (e.g. gene, DNA), yet they were still less familiar with modern concepts such as “personalized medicine,” “pharmacogenomics,” or “biomarkers” [25]. Furthermore, self‐reported numeracy has been seen to correlate with a familiarity and knowledge of genetic terms [19]. However, health literacy and numeracy measures have had only weak or moderate correlation with various measures of genomic literacy [22]. Further research is needed to explore the connection between health literacy and genomic literacy.

The aim of this longitudinal feasibility pilot study was to assess the health and genomic literacy of participants at risk of Type 2 diabetes, to better understand the factors influencing health and genomic literacy, as well as the relationship between these two literacy perspectives.

## 2. Materials and Methods

### 2.1. Study Setting and Sampling

The study recruited Finnish speaking individuals aged 18 to 64 who were at elevated risk for Type 2 diabetes, had prediabetes, or had received diagnosis of Type 2 diabetes within the previous year. More specific eligibility criteria were a Finnish diabetes risk score (FINDRISC) above 12 [26], a prediabetes defined according to WHO criteria as impaired fasting glucose (FPG) 6.1–6.9 mmol/L or impaired glucose tolerance (IGT) 2‐h plasma glucose 7.8–11 mmol/L [27], or physician‐diagnosed Type 2 diabetes. In this study, no distinction was made among participants with elevated risk based on whether they already had a diagnosis or not.

Recruitment took place either during their appointments with a public health nurse due to a known elevated diabetes risk or while visiting the health center for other reasons. During these visits, the inclusion criteria for the study were evaluated. Additionally, one public research bulletin was posted in a local Facebook group maintained by the townspeople of the city.

Recruitment took place between June and December 2022 and ended once the intended target of 60 participants for this pilot study was reached. The informed consent process was managed by a single research nurse (a public health nurse), and participants provided written informed consent before entering the study. All participants (*n* = 60) completed the baseline surveys. All participants (*n* = 60) received their PRS results approximately 1 year after enrollment, and the postsurveys were distributed 2 weeks later. A total of 49 participants (81.7%) returned the completed postsurveys.

### 2.2. Study Interventions

After the baseline surveys, blood samples were collected to determine participants’ PRS for Type 2 diabetes, hemoglobin A1c, plasma lipids, and blood count for the assessment of hemoglobin A1c reliability. Oral glucose tolerance test was performed for participants yet not having a prediabetes or Type 2 diabetes diagnoses. These surveys and tests were carried out from June 2022 to December 2022 after recruitment. The PRS results were analyzed in larger batches; therefore, PRS results were disclosed to all participants toward the end of the research period.

Participants received a remote reception (video call) during May–June 2023. In the video call, a physician presented the PRS results and follow‐up plans, and a public health nurse continued with individual lifestyle counseling. The PRS result was visually represented by a Gaussian curve. At the peak, the PRS was 0, indicating the average risk in the population. PRS values are generally distributed between −4 and 4, with a higher number indicating a higher risk. The participant could therefore understand whether their PRS indicated a higher or lower risk compared to the general population. The discussion focused on the PRS, the risk of Type 2 diabetes, and the disease status. Lifestyle counseling was tailored to individual needs. The counseling highlighted that, in addition to age, lifestyle and genetic factors influence the risk of developing Type 2 diabetes and that overall risk can be modified by lifestyle factors. A healthy lifestyle is supported by, among other things, good nutrition, exercise, and sleep. The video calls lasted approximately 45 min. Two weeks after each video call, participants received the postsurveys, which they completed at home and returned electronically or by mail. Consequently, the follow‐up period for assessing change was relatively short.

Participants also had access to a website [https://ehkaisetyyppi2.fi] with information on the prevention of Type 2 diabetes, although technical limitations made it impossible to assess website usage or time spent on the site.

### 2.3. Instruments

#### 2.3.1. Health Literacy

Health literacy was measured using both the Everyday Health Information Literacy (EHIL) instrument [28], and a shortened version of the European Health Literacy Survey Questionnaire (HLS‐EU‐Q) [29], which had been translated in an earlier study into Finnish and then back into English [30].

The EHIL included 10 items, each rated on a 5‐point Likert scale (from “Strongly Disagree” to “Strongly Agree”). Some previous studies have used the EHIL as a 10‐item sum variable, with negatively worded items reverse‐coded (Items 4, 5, 8, and 10), resulting in a possible score range of 10–50 [31–33]. The EHIL instrument has also been proposed for use as three different factors, namely, *motivation* (Items 1, 2, 3, and 9), *confidence* (Items 4, 5, and 10), and *evaluation* (Items 6 and 7) [28]. Hirvonen et al. [33] added Item 8 within the other Items 4, 5, and 10. In this feasibility pilot study, the small sample size prevented conducting a factor analysis itself; however, these factors were nevertheless applied in the present study. Sum scores were calculated for each factor. The instrument has previously been used among participants in a health‐related risk group [31]. Participants responded in Finnish, the instrument’s original language.

The HLS‐EU‐Q16 consisted of 16 items rated on a 4‐point Likert scale (from 1 = “Very Difficult” to 4 = “Very Easy”), with an additional fifth option, “I don’t know” (coded as missing), resulting a minimum score of 16 and a maximum of 64. A sum variable was constructed based on the respondents’ mean scores, 1–4 for each respondent. These mean scores were converted to a 0–50 scale for those who had answered at least 80% of the items (13 items), using a formula: Index = (mean − 1) × (50/3). Using these index scores, health literacy levels were categorized as follows: inadequate (0–25), problematic (> 25–33), sufficient (> 33–42), and excellent (> 42–50) health literacy [34].

#### 2.3.2. Genomic Literacy

While health literacy was assessed by personal experience, genomic literacy was assessed by a knowledge test. In this study, genomic literacy was operationalized as a combination of Sections A, B, and C. Sections A and B were assessed using a shortened instrument developed by Jallinoja and Aro [35] to evaluate knowledge of genes and heredity. Originally, Section A included 16 items, with answer options: correct, not correct, or I don’t know [35]. In this study, we used 10 of these items since they were also used in the Haukkala et al. [36] study. Section B included five original items. However, the original version was related to chronic diseases, whereas here, the items focused on Type 2 diabetes. The one original phrase “*The person has the disease in a mild form*” was revised to *“The person’s blood sugar is slightly elevated, but they do not yet have diabetes”* to reflect the context of Type 2 diabetes. Also, compared to the original version, a sixth question item was added: “*What does it mean if a person has a low genetic predisposition to developing type 2 diabetes?”,* followed by the statement: *“Unhealthy lifestyle choices cannot increase the risk of disease,”* with the same answer options: correct, not correct, or I don’t know.

Section C addressed genomic numeracy by assessing the understanding of the risk result of a genomic test. Section C begins with the sentence: *“If you have received a genomic test result indicating that you have a 7% risk of developing type 2 diabetes within the next 10 years based on your current lifestyle*,*”* continuing with five different statements. Each could be answered with correct, not correct, or I don’t know. These question items were developed during the feasibility study, as numeracy is a component of health literacy and becomes particularly important in genomic literacy, where communicating results requires an understanding of risk and probability [13]. Section C items were based on a statement indicating one way to represent the risk of developing Type 2 diabetes based on the PRS result. These items operationalized the probability and interpretation of the result and were reviewed by experts (a geneticist, a genomics researcher, and a physician).

Jallinoja and Aro [35] titled Section A “knowledge about genes and heredity,” assessing understanding of genetic concepts; Section B “knowledge about susceptibility,” focusing on the application and use of knowledge; and, in this study, Section C was termed “genomic numeracy,” addressing numeracy and the ability to interpret results. When only one section was analyzed at a time, it was indicated, for example, as “genomic literacy, Section B.”

The sum score for Section A was constructed by assigning 1 point for each correct answer and 0 points if the response was incorrect or “I don’t know,” with a maximum score of 10 points. We used only the items from Section A to create a separate sum variable, since these items have been combined in earlier studies [36], although originally a set was comprised of 16 items [35]. Section C was also analyzed separately, with a maximum score of 5 points. Section B was not included in the sum variables, as some of its questions were newly developed. This exclusion also enabled a better comparability of Section A with previous studies.

### 2.4. Data Analysis

Data were described using frequencies and percentages for categorical variables (gender, education, survey items), means and standard deviations (SD) for continuous variables with normal distributions (sum of EHIL, and HLS‐EU‐Q16 index), and medians, lower (*Q*_1_), and upper (*Q*_3_) quartiles for continuous variables with skewed distribution (sum of genomic literacy, Sections A and C, and factors of EHIL). Minimum and maximum values were reported with the median age. The factors of the EHIL (motivation, confidence, evaluation) were all assessed as skewed due to consistency.

The explanatory variables used were gender, age, and level of education. Since only one participant identified as “other” in terms of gender, this response was coded as missing. Consequently, statistical tests related to gender were conducted for two unpaired groups. Statistical tests related to education were conducted based on baseline educational level at both measurement points, while a 1‐year increase in age was accounted for at the second measurement point.

As a longitudinal design, paired *t* test was used when comparing two normally distributed paired groups (EHIL and HLS‐EU‐Q16 index) between baseline and postsurvey time points. Similarly, if the distribution was skewed, the Wilcoxon test was used (genomic literacy, Sections A and C, motivation, confidence, and evaluation [37]. Paired comparisons were performed only for cases with valid values at both time points, whereas other analyses included all cases with available data.

Health literacy instruments EHIL (including factors: motivation, confidence, evaluation), HLS‐EU‐Q16, and genomic literacy, Sections A and C, were examined in relation to the explanatory variables. Unpaired *t* test (if the variable was normally distributed) and the Mann–Whitney *U* test (if there was a skewed distribution) were used to compare two groups (gender). Pearson correlation was used for normal continuous variables (EHIL, HLS‐EU‐Q16 index) and Spearman correlation when at least one of the continuous variables was skewed (age, EHIL factors, genomic literacy, Sections A and C). One‐way ANOVA (if the variable was normally distributed) and Kruskal–Wallis test (if there was a skewed distribution) were applied to compare three or more independent groups (level of education) [37].

The internal consistency of the EHIL and HLS‐EU‐Q16 was measured with Cronbach’s alpha. Statistical significance was determined at the *p* < 0.05 level. The correlation was considered as weak (0.00–0.29), moderate (0.30–0.49), and strong (0.5–1.00) [37]. The data were analyzed using SPSS Statistics, Version 30.

### 2.5. Ethical Considerations

This study protocol was reviewed and approved by the Regional Ethics Committee of the Expert Responsibility area of Tampere University Hospital (Approval No. R22018). Participants provided written informed consent for participation.

Although all participants were initially aware of their elevated risk of Type 2 diabetes, personal counseling also allowed participants’ concerns to be addressed during the PRS disclosure. Personal counseling was also important from an equality perspective. Participants had the opportunity to discuss, refine, and clarify the results they received, and the counseling could be tailored to the individual, taking into account participants’ health literacy and understanding, particularly among those with a lower educational level.

## 3. Results

### 3.1. Characteristics of the Sample

Among the participants, 68.3% (*n* = 41) were women, 30.0% (*n* = 18) men, and 1.7% (*n* = 1) selected “other” for gender. One year later, 77.6% (*n* = 38) women and 22.4% (*n* = 11) men completed the postsurveys. At baseline, women were on average 54.0 years old (min 24, max 64), and men were 58.5 years old (min 43, max 63) (Table 1).

**TABLE 1:** Participant demographics.

Background variables	Baseline survey (*n* = 60)			Postsurvey (*n* = 49)		
	*n*	%	Md age (min, max)	*n*	%	Md age (min, max)
Gender						
Female	41	68.3	54.0 (24, 64)	38	77.6	56.0 (25, 65)
Male	18	30.0	58.5 (43, 63)	11	22.4	58.0 (44, 64)
Other	1	1.7	—	—	—	—
All	60	100	55.0 (24, 64)	49	100	56.0 (25, 65)
Education^a^						
Primary education or no degree	11	18.3		8	16.3	
Secondary education	23	38.3		19	38.8	
Bachelor’s degree	11	18.3		10	20.4	
Master’s degree or higher	15	25.0		12	24.5	
Work situation						
Full‐time work	21	35.0		17	34.7	
Part‐time work	6	10.0		5	10.2	
Unemployed	19	31.7		11	22.4	
Pension	9	15.0		13	26.5	
Parental leave	2	3.3		—	—	
Student	—	—		2	4.1	
Practical training	1	1.7		—	—	
Sickness allowance	1	1.7		1	2.0	
Other	1	1.7		—	—	

^a^The education level was based on the baseline survey responses at both time points.

At baseline, 31.7% of participants were unemployed, which is considerably higher than the average for people aged 55–64 in Finland, and in 2022, the unemployment rate for this age group was 7.3% [38]. At postsurvey time point, the trend seemed to be moving toward retirement. In terms of education, 81.6% had at least secondary education, which is generally lower than seen in Finland among the 50–64 age groups in 2022 (85.1%–87.0%) [39].

### 3.2. Health Literacy—EHIL

Responses to the EHIL instrument are shown in Table 2. The internal consistency of the EHIL scale approached acceptable levels in the baseline, with a Cronbach alpha of 0.67, which is slightly below the threshold of 0.70 for moderate internal reliability [37]. In the time of the postsurveys, the Cronbach alpha was 0.70.

**TABLE 2:** Response distribution of the EHIL questionnaire in the baseline and postsurveys.

The Everyday Health Information Literacy (EHIL)	Baseline *n* (%) (*n* = 60)					Postsurvey *n* (%) (*n* = 49)				
	1^a^	2	3	4	5	1	2	3	4	5
1. It is important to be informed about health issues.	1 (1.7)	—	—	17 (28.3)	42 (70.0)	—	—	1 (2.0)	17 (34.7)	31 (63.3)
2. I know where to seek health information.	1 (1.7)	2 (3.3)	6 (10.0)	32 (53.3)	19 (31.7)	—	—	2 (4.1)	22 (44.9)	25 (51.0)
3. I like to get health information from a variety of sources.	1 (1.7)	1 (1.7)	6 (10.0)	26 (43.3)	26 (43.3)	—	1 (2.0)	2 (4.1)	21 (42.9)	25 (51.0)
4. It is difficult to find health information from printed sources (magazines and books).	10 (16.7)	26 (43.3)	15 (25.0)	7 (11.7)	2 (3.3)	11 (22.4)	18 (36.7)	7 (14.3)	11 (22.4)	2 (4.1)
5. It is difficult to find health information from the internet.	28 (46.7)	22 (36.7)	5 (8.3)	5 (8.3)	—	22 (44.9)	19 (38.8)	4 (8.2)	4 (8.2)	—
6. It is easy to assess the reliability of health information in printed sources (magazines and books).	2 (3.3)	16 (26.7)	16 (26.7)	22 (36.7)	4 (6.7)	—	16 (32.7)	9 (18.4)	19 (38.8)	5 (10.2)
7. It is easy to assess the reliability of health information on the internet.	3 (5.0)	17 (28.3)	14 (23.3)	21 (35.0)	5 (8.3)	—	23 (46.9)	10 (20.4)	12 (24.5)	4 (8.2)
8. Health‐related terminology and statements are often difficult to understand.	3 (5.0)	18 (30.0)	11 (18.3)	24 (40.0)	4 (6.7)	6 (12.2)	17 (34.7)	9 (18.4)	13 (26.5)	4 (8.2)
9. I apply health‐related information to my own life and/or that of people close to me.	1 (1.7)	1 (1.7)	10 (16.7)	33 (55.0)	15 (25.0)	—	3 (6.1)	6 (12.2)	27 (55.1)	13 (26.5)
10. It is difficult to know who to believe in health issues.	11 (18.3)	23 (38.3)	13 (21.7)	12 (20.0)	1 (1.7)	6 (12.2)	17 (34.7)	7 (14.3)	17 (34.7)	2 (4.1)

^a^a 5‐point Likert scale: 1 = Strongly Disagree, 2 = Disagree, 3 = Neither Agree nor Disagree, 4 = Agree, 5 = Strongly Agree.

In the baseline survey, the average EHIL sum score was 37.8 (SD 4.0, *n* = 49), and during the postsurveys, the average was 37.8 (SD 4.9, *n* = 49), showing no statistically significant difference between the baseline and postsurvey time points (*p* = 0.952) (Table 3).

**TABLE 3:** Change in health literacy and genomic literacy, Sections A and C, during intervention.

Health literacy and genomic literacy instruments	Baseline	Postsurvey	Paired comparison test
EHIL (*n* = 49) mean (SD)	37.8 (4.0)	37.8 (4.9)	*p* = 0.952^a^
Motivation median (Q1, Q3)	17.0 (16.0, 18.0)	18.0 (16.0, 19.0)	*p* = 0.107^b^
Confidence median (Q1, Q3)	15.0 (13.0, 16.0)	14.0 (12.0, 16.0)	*p* = 0.409^b^
Evaluation median (Q1, Q3)	6.0 (5.0, 8.0)	6.0 (4.0, 8.0)	*p* = 0.647^b^
The HLS‐EU‐Q16 index (*n* = 44) mean (SD)	38.8 (6.5)	37.6 (6.3)	*p* = 0.080^a^
Genomic literacy, Section A (*n* = 49) median (Q1, Q3)	8.0 (7.0, 9.0)	9.0 (7.0, 9.0)	**p** ** = 0.012** ^b^
Genomic literacy, Section C (*n* = 49) median (Q1, Q3)	4.0 (4.0, 5.0)	4.0 (4.0, 5.0)	*p* = 0.964^b^

*Note:* Statistically significant marked in bold.

^a^Paired *t* test.

^b^Wilcoxon test.

There were no statistically significant differences in EHIL scores between genders at baseline (*p* = 0.085). After the intervention, men scored an average of 34.8 (SD 3.4, *n* = 11), and women scored 38.6 (SD 5.0, *n* = 38). This time, there was a statistically significant difference, with women scoring higher (*p* = 0.022). There was a weak negative correlation between EHIL scores and age, whereas age increased, and EHIL scores decreased (*r* = −0.27, *p* = 0.034). A similar trend was also observed in the postsurveys (*r* = −0.32, *p* = 0.024) (Table 4).

**TABLE 4:** Relationship between health literacy and genomic literacy, Sections A and C, and explanatory variables.

Explanatory variables	Health literacy and genomic literacy instruments						
	EHIL mean (SD)	Motivation median (*Q*_1_, *Q*_3_)	Confidence median (*Q*_1_, *Q*_3_)	Evaluation median (*Q*_1_, *Q*_3_)	HLS‐EU‐Q16 index mean (SD)	Genomic literacy, Section A median (*Q*_1_, *Q*_3_)	Genomic literacy, Section C median (*Q*_1_, *Q*_3_)
Baseline (*n* = 60)	37.5 (4.7)	17.0 (16.0, 18.0)	15.0 (12.0, 16.0)	6.0 (5.0, 8.0)	38.5 (6.8) (*n* = 52)	8.0 (7.0, 9.0)	4.0 (3.5, 5.0)
Gender	*p* = 0.085^a^	*p* = 0.090^b^	*p* = 0.300^b^	*p* = 0.331^b^	*p* = 0.071^a^ (*n* = 51)	*p* = 0.609^b^	*p* = 0.882^b^
Men (*n* = 18)	36.0 (4.7)	17.0 (15.0, 18.0)	14.0 (11.0, 16.0)	6.0 (4.0, 8.0)	35.9 (5.7) (*n* = 15)	8.5 (7.0, 10.0)	4.0 (4.0, 5.0)
Women (*n* = 41)	38.3 (4.6)	18.0 (16.0, 19.0)	15.0 (13.0, 16.0)	6.0 (5.0, 8.0)	39.7 (7.0) (*n* = 36)	8.0 (7.0, 9.0)	4.0 (3.0, 5.0)
Age	*r* = −0.27, **p** ** = 0.034** ^c^	*r* = −0.10, *p* = 0.445^c^	*r* = −0.28, **p** ** = 0.033** ^c^	*r* = −0.17, *p* = 0.190^c^	*r* = −0.15, *p* = 0.295^c^	*r* = −0.35, **p** ** = 0.006** ^c^	*r* = −0.17, *p* = 0.192^c^
Education	**p** ** = 0.005** ^d^, ^f^	*p* = 0.265^e^	**p** ** = 0.014** ^e^, ^g^	*p* = 0.082^e^	**p** ** = 0.037** ^d^, ^h^	**p** ** = 0.001** ^e^, ^i^	*p* = 0.123^e^
Primary (*n* = 11)	33.2 (3.0)	17.0 (15.5, 18.0)	11.0 (9.5, 13.5)	5.0 (4.0, 6.0)	32.7 (8.0) (*n* = 9)	7.0 (6.0, 8.5)	4.0 (2.5, 5.0)
Secondary (*n* = 23)	37.8 (4.4)	17.0 (16.0, 18.0)	15.0 (13.0, 17.0)	6.0 (6.0, 8.0)	39.2 (7.5) (*n* = 18)	7.0 (6.0, 8.5)	4.0 (3.0, 5.0)
Bachelor’s (*n* = 11)	39.5 (5.4)	18.0 (15.5, 18.5)	15.0 (13.5, 16.5)	8.0 (6.0, 8.0)	41.0 (5.1) (*n* = 10)	8.0 (7.0, 8.5)	5.0 (4.0, 5.0)
Master’s or higher (*n* = 15)	38.7 (4.3)	18.0 (16.0, 19.0)	15.0 (13.5, 16.0)	6.0 (4.0, 7.5)	39.3 (4.5) (*n* = 15)	10.0 (9.0, 10.0)	5.0 (4.0, 5.0)
Postsurveys (*n* = 49)	37.8 (4.9)	18.0 (16.0, 19.0)	14.0 (12.0, 16.0)	6.0 (4.0, 8.0)	37.2 (6.8) (*n* = 45)	9.0 (7.0, 9.0)	4.0 (4.0, 5.0)
Gender	**p** ** = 0.022** ^a^	*p* = 0.052^b^	*p* = 0.073^b^	*p* = 0.288^b^	**p** ** = 0.010** ^a^ (*n* = 45)	*p* = 0.630^b^	*p* = 0.165^b^
Men (*n* = 11)	34.8 (3.4)	16.0 (15.5, 18.0)	13.0 (11.0, 13.5)	5.0 (4.0, 7.0)	32.1 (6.1) (*n* = 9)	9.0 (8.5, 9.0)	5.0 (4.0, 5.0)
Women (*n* = 38)	38.6 (5.0)	18.0 (17.0, 19.0)	14.5 (13.0, 17.0)	6.0 (5.0, 8.0)	38.5 (6.4) (*n* = 36)	9.0 (7.0, 10.0)	4.0 (3.0, 5.0)
Age	*r* = −0.32, **p** ** = 0.024** ^c^	*r* = −0.24, *p* = 0.104^c^	*r* = −0.22,*p* = 0.139^c^	*r* = −0.25, *p* = 0.089^c^	*r* = −0.035, *p* = 0.822^c^	*r* = −0.29, **p** ** = 0.047** ^c^	*r* = −0.14, *p* = 0.327^c^
Education	**p** ** = 0.005** ^d^, ^j^	*p* = 0.172^e^	**p** ** = 0.005** ^e^, ^k^	*p* = 0.311^e^	**p** ** = 0.041** ^d^, ^l^	*p* = 0.227^e^	*p* = 0.247^e^
Primary (*n* = 8)	34.3 (3.7)	18.0 (15.5, 19.0)	10.0 (9.0, 13.5)	6.0 (4.5, 8.0)	31.7 (7.7) (*n* = 7)	8.5 (7.0, 9.0)	5.0 (4.0, 5.0)
Secondary (*n* = 19)	36.8 (4.8)	17.0 (16.0, 19.0)	13.0 (12.0, 16.0)	5.0 (4.0, 6.5)	36.9 (6.0) (*n* = 16)	8.0 (7.0, 9.0)	4.0 (3.0, 5.0)
Bachelor’s (*n* = 10)	37.9 (4.8)	16.5 (16.0, 19.0)	13.5 (13.0, 16.0)	6.5 (6.0, 8.0)	37.1 (7.5) (*n* = 10)	8.5 (8.0, 10.0)	4.0 (3.0, 5.0)
Master’s or higher (*n* = 12)	41.5 (3.8)	19.0 (18.0, 19.5)	16.0 (15.0, 17.5)	7.0 (4.5, 8.0)	40.8 (4.6) (*n* = 12)	9.0 (9.0, 10.0)	4.5 (4.0, 5.0)

*Note:* Statistically significant marked in bold.

^a^Unpaired *t* test.

^b^Mann–Whitney *U* test.

^c^Spearman’s correlation.

^d^One‐way ANOVA.

^e^Kruskal–Wallis test.

^f^Baseline EHIL: primary–secondary *p* = 0.031, primary–bachelor’s degree *p* = 0.008, primary–master’s degree or higher *p* = 0.014.

^g^Baseline confidence: primary–secondary *p* = 0.031, primary–bachelor’s degree *p* = 0.037, primary–master’s degree or higher *p* = 0.034.

^h^Baseline HLS‐EU‐Q16 index: primary–bachelor’s degree *p* = 0.046.

^i^Baseline genomic literacy A: primary–master’s degree or higher *p* = 0.014, secondary–master’s degree or higher *p* = 0.001.

^j^Postsurvey EHIL: primary–master’s degree or higher *p* = 0.005, secondary–master’s degree or higher *p* = 0.038.

^k^Postsurvey confidence: primary–master’s degree or higher *p* = 0.002.

^l^Postsurvey HLS‐EU‐Q16: primary–master’s degree or higher *p* = 0.028.

With primary education, the average EHIL score was 33.2 (SD 3.0, *n* = 11), secondary level 37.8 (SD 4.4, *n* = 23), bachelor’s degree 39.45 (SD 5.4, *n* = 11), and with a master’s degree or higher, it was 38.7 (SD 4.3, *n* = 15). There was a statistically significant difference between education levels (*p* = 0.005). Those who had only primary education had statistically significantly lower EHIL scores than those who had secondary education (*p* = 0.031), bachelor’s degree (*p* = 0.008), or master’s degree or higher (*p* = 0.014). There was no statistically significant difference between the other educational groups. After intervention, among those with primary education, the average EHIL score was 34.3 (SD 3.7, *n* = 8), secondary education 36.8 (SD 4.8, *n* = 19), bachelor’s degree 37.9 (SD 4.8, *n* = 10), and those with master’s level or higher, it was 41.5 (SD 3.8, *n* = 12). There was also a statistically significant difference between education levels (*p* = 0.005), but now only those with master’s degree or higher scored statistically significant higher scores than those who had primary (*p* = 0.005) and secondary (*p* = 0.038) education (Table 4).

#### 3.2.1. Motivation, Confidence, and Evaluation

When motivation, confidence, and evaluation were assessed as part of EHIL over the intervention, there were no significant differences with motivation (*p* = 0.107), confidence (*p* = 0.409), and evaluation (*p* = 0.647) between the baseline and postsurvey time points (Table 3). Cronbach’s alpha values for the EHIL factors at baseline and postsurvey were 0.60 and 0.78 for motivation, 0.62 and 0.65 for confidence, and 0.75 and 0.77 for evaluation.

Gender had no statistically significant association on motivation at the beginning (*p* = 0.090), but at the end of intervention, the difference in motivation between women and men approached statistical significance, with women being more motivated (*p* = 0.052). Motivation did not correlate with age at either the baseline or postsurvey time point (*p* = 0.445; *p* = 0.104), and there was no statistically significant difference in motivation between different education levels at either point (*p* = 0.265; *p* = 0.172) (Table 4).

Confidence did not differ between genders at either time point (*p* = 0.300; *p* = 0.073). There was a weak negative correlation with confidence and age where confidence decreased as age increased (*r* = −0.28, *p* = 0.033), but not at the time of postsurvey (*p* = 0.139). In addition, confidence was better at higher levels of education than with primary education (primary–secondary *p* = 0.031, primary–bachelor’s degree *p* = 0.037, primary–master’s degree or higher *p* = 0.034). At the postsurvey, a difference was seen only between the primary education level and a master’s degree or higher (*p* = 0.002) (Table 4).

Evaluation did not differ between genders (*p* = 0.331; *p* = 0.288). There was no correlation between evaluation and age (*p* = 0.190; *p* = 0.089), and evaluation did not significantly differ between education levels (*p* = 0.082; *p* = 0.311) (Table 4).

### 3.3. Health Literacy—HLS‐EU‐Q16

Responses to the HLS‐EU‐Q16 questionnaire are shown in Table 5. The internal consistency of the HLS‐EU‐Q16 questionnaire was good, with a Cronbach alpha of 0.88 at both baseline and postsurvey time points. The HLS‐EU‐Q16 index variable showed an average score of 38.8 (SD 6.5, *n* = 44) at baseline and 37.6 (SD 6.3, *n* = 44) at the postsurvey time point, showing no significant difference (*p* = 0.080) (Table 3). Both baseline and postsurvey scores indicated a sufficient health literacy level (> 33–42).

**TABLE 5:** Response distribution of the HLS‐EU‐Q16 questionnaire in the baseline and postsurveys.

HLS‐EU‐Q16	Baseline *n* (%) (*n* = 60)					Postsurvey *n* (%) (*n* = 49)				
On a scale from very easy to very difficult, how easy would you say it is to …	1^a^	2	3	4	5	1	2	3	4	5
1. Find information on treatment of illnesses that concern you?	1 (1.7)	4 (6.7)	31 (51.7)	21 (35.0)	3 (5.0)	—	4 (8.2)	24 (49.0)	19 (38.8)	2 (4.1)
2. Find out where to get professional help when you are ill?	1 (1.7)	11 (18.3)	22 (36.7)	25 (41.7)	1 (1.7)	—	8 (16.3)	28 (57.1)	13 (26.5)	—
3. Understand what your doctor says to you?	—	4 (6.7)	36 (60.0)	20 (33.3)	—	1 (2.0)	7 (14.3)	22 (44.9)	18 (36.7)	1 (2.0)
4. Understand your doctor’s or pharmacist’s instruction on how to take a prescribed medicine?	—	2 (3.3)	12 (20.0)	46 (76.7)	—	—	1 (2.0)	18 (36.7)	29 (59.2)	1 (2.0)
5. Judge when you may need to get a second opinion from another doctor?	3 (5.0)	15 (25.0)	22 (36.7)	10 (16.7)	10 (16.7)	2 (4.1)	21 (42.9)	12 (24.5)	6 (12.2)	8 (16.3)
6. Use information the doctor gives you to make decisions about your illness?	—	4 (6.7)	31 (51.7)	23 (38.3)	2 (3.3)	—	6 (12.2)	22 (44.9)	17 (34.7)	4 (8.2)
7. Follow instructions from your doctor or pharmacist?	—	—	14 (23.3)	45 (75.0)	1 (1.7)	—	2 (4.1)	15 (30.6)	32 (65.3)	—
8. Find information on how to manage mental health problems like stress or depression?	4 (6.7)	7 (11.7)	24 (40.0)	21 (35.0)	4 (6.7)	2 (4.1)	4 (8.2)	25 (51.0)	13 (26.5)	5 (10.2)
9. Understand health warnings about behavior such as smoking, low physical activity, and drinking too much?	—	1 (1.7)	10 (16.7)	48 (80.0)	1 (1.7)	—	—	10 (20.4)	39 (79.6)	—
10. Understand why you need health screenings?	—	2 (3.3)	14 (23.3)	43 (71.7)	1 (1.7)	—	—	7 (14.3)	41 (83.7)	1 (2.0)
11. Judge if the information on the health risks in the media is reliable?	3 (5.0)	11 (18.3)	24 (40.0)	14 (23.3)	8 (13.3)	2 (4.1)	14 (28.6)	21 (42.9)	10 (20.4)	2 (4.1)
12. Decide how you can protect yourself from illness based on information in the media?	1 (1.7)	16 (26.7)	23 (38.3)	11 (18.3)	9 (15.0)	2 (4.1)	15 (30.6)	23 (46.9)	6 (12.2)	3 (6.1)
13. Find out about activities that are good for your mental well‐being?	—	8 (13.3)	28 (46.7)	23 (38.3)	1 (1.7)	1 (2.0)	5 (10.2)	25 (51.0)	15 (30.6)	3 (6.1)
14. Understand advice on health from family members or friends?	2 (3.3)	6 (10.0)	25 (41.7)	22 (36.7)	5 (8.3)	—	2 (4.1)	25 (51.0)	19 (38.8)	3 (6.1)
15. Understand information in the media on how to get healthier?	—	11 (18.3)	27 (45.0)	13 (21.7)	9 (15.0)	1 (2.0)	12 (24.5)	24 (49.0)	8 (16.3)	4 (8.2)
16. Judge which everyday behavior is related to your health?	—	4 (6.7)	23 (38.3)	32 (53.3)	1 (1.7)	2 (4.1)	3 (6.1)	21 (42.9)	23 (46.9)	—

^a^4‐point Likert scale: 1 = Very Difficult, 2 = Fairly Difficult, 3 = Fairly Easy, 4 = Very Easy, 5 = I Don’t Know.

In the baseline survey, there were no gender differences between HLS‐EU‐Q16 index scores (*p* = 0.071). However, a statistically significant difference was observed in the postsurvey (*p* = 0.010), with men scoring an average of 32.1 (SD 6.1, *n* = 9) and women scoring higher 38.5 (SD 6.4, *n* = 36). There was no significant correlation between HLS‐EU‐Q16 index and age at either baseline or postsurvey point (*p* = 0.295; *p* = 0.822) (Table 4).

A statistically significant association was found between education groups and HLS‐EU‐Q16 index (*p* = 0.037). Participants with a primary level education scored an average of 32.7 (SD 8.0, *n* = 9), whereas those with a bachelor’s degree scored significantly higher (*p* = 0.046) with an average of 41.0 (SD 5.1, *n* = 10). After intervention, there was also a significant difference between education groups (*p* = 0.041). Participants with a primary level education scored an average of 31.7 (SD 7.7, *n* = 7), while those with a master’s level education or higher scored an average of 40.8 (SD 4.6, *n* = 12), which was statistically significantly higher compared to the primary level (*p* = 0.028). Although no statistically significant differences were found between the other education levels, there was a trend of increasing scores with higher educational attainment (Table 4). Both at baseline and postsurvey, participants with only primary level education scored below 33, which classified health literacy as *problematic* (> 25–33), whereas participants with other education levels consistently remained within the *sufficient* range (> 33–42).

### 3.4. Genomic Literacy

#### 3.4.1. Section A

Items from Section A, which addressed concepts such as *“Different body parts include different genes”* (48.3% correct), *“Genes are inside cells”* (55% correct), and *“A gene is a part of a chromosome”* (60.0% correct), were the most difficult for participants, excluding *“A gene is a disease,”* which was 95.0% correct. In contrast, questions related to the onset of disease and heredity received more correct answers: *“The onset of certain disease is due to genes, environment and lifestyle”* (100% correct), *“Healthy parents can have a child with a hereditary disease”* (91.7% correct), and *“The carrier of a disease gene may be completely healthy”* (90.0% correct) (Table 6).

**TABLE 6:** Responses to genomic literacy, Sections A, B, and C, in the baseline and postsurveys.

	Baseline *n* (%) (*n* = 60)	Postsurvey *n* (%) (*n* = 49)
	Correct responses	Correct responses
*A. Knowledge of genes and heredity*		
Statements:		
1. The onset of certain diseases is due to genes, environment, and lifestyle. (Correct)	60 (100)	48 (98.0)
2. A gene is a disease. (Not correct)	57 (95.0)	48 (98.0)
3. Healthy parents can have a child with a hereditary disease. (Correct)	55 (91.7)	47 (95.9)
4. The carrier of a disease gene may be completely healthy. (Correct)	54 (90.0)	47 (95.9)
5. All serious diseases are hereditary. (Not correct)	55 (91.7)	45 (91.8)
6. The child of a disease gene carrier is always also a carrier of the same disease gene. (Not correct)	44 (73.3)	37 (75.5)
7. A gene is a piece of DNA. (Correct)	44 (73.3)	43 (87.8)
8. Genes are inside cells. (Correct)	33 (55.0)	36 (73.5)
9. A gene is a part of a chromosome. (Correct)	36 (60.0)	29 (59.2)
10. Different body parts include different genes. (Not correct)	29 (48.3)	24 (49.0)

*B. Knowledge about susceptibility*		
What does it mean if a person has a hereditary susceptibility to Type 2 diabetes?		
1. There is no way to prevent the onset of the Type 2 diabetes. (Not correct)	56 (93.3)	48 (98.0)
2. The person has a higher‐than‐average risk of developing Type 2 diabetes. (Correct)	57 (95.0)	45 (91.8)
3. The person’s children may also be susceptible to Type 2 diabetes. (Correct)	54 (90.0)	45 (91.8)
4. It is possible to prevent the onset of the Type 2 diabetes by means of appropriate lifestyle. (Correct)	58 (96.7)	48 (98.0)
5. The person’s blood sugar is slightly elevated, but they do not yet have diabetes. (Not correct)	20 (33.3)	16 (32.7)
What does it mean if a person has a low hereditary susceptibility to Type 2 diabetes?		
6. Poor lifestyle choices cannot increase their risk of developing the disease. (Not correct)	52 (86.7)	40 (81.6)

*C. Understanding the risk result of a genomic test*		
If you have received a genomic test result indicating that you have a 7% risk of developing Type 2 diabetes within the next 10 years based on your current lifestyle.		
1. Seven out of 100 people like you will develop Type 2 diabetes within 10 years. (Correct)	38 (63.3)	36 (73.5)
2. You can reduce your 7% risk of developing the disease by adopting a healthy lifestyle. (Correct)	48 (80.0)	41 (83.7)
3. You cannot reduce your 7% risk of developing the disease even if you follow recommended lifestyle guidelines. (Not correct)	51 (85.0)	44 (89.8)
4. Your risk may increase beyond 7% if your lifestyle worsens in the future. (Correct)	56 (93.3)	43 (87.8)
5. You will develop Type 2 diabetes at some point in your life. (Not correct)	46 (76.7)	33 (67.3)

There was a statistically significant difference between baseline and postsurvey scores in Section A (*p* = 0.012), comparing a median of 8.0 (*Q*_1_ 7.0, *Q*_3_ 9.0, *n* = 49) at baseline and 9.0 (*Q*_1_ 7.0, *Q*_3_ 9.0, *n* = 49) at the postsurvey (Table 3). Effect size (*r* = 0.361) was considered medium [40].

There was no statistically significant difference in genomic literacy, Section A, between genders at either baseline or the postsurvey time point (*p* = 0.609; *p* = 0.630), but the age showed a moderate negative correlation at baseline, indicating that younger participants had better scores (*r* = −0.35, *p* = 0.006, *n* = 60). After intervention, the association was little less pronounced (*r* = −0.29, *p* = 0.047, *n* = 49) (Table 4).

The primary education level scored a median of 7.0 (*Q*_1_ 6.0, *Q*_3_ 8.5, *n* = 11) in Section A, the secondary level also scored 7.0 (*Q*_1_ 6.0, *Q*_3_ 8.5, *n* = 23), those with a bachelor’s degree scored 8.0 (*Q*_1_ 7.0, *Q*_3_ 8.5, *n* = 11), and those with a master’s degree or higher scored 10.0 (*Q*_1_ 9.0, *Q*_3_ 10.0, *n* = 15). There was a statistically significant difference between education levels (*p* = 0.001), with participants holding a master’s degree or higher scoring significantly higher than those with primary (*p* = 0.014) or secondary (*p* = 0.001) education. At the postsurvey, there was no longer a statistically significant difference between education levels (*p* = 0.227) (Table 4).

#### 3.4.2. Section B

Section B addressed hereditary susceptibility (Table 6). The first question *“What does it mean if a person has a hereditary susceptibility to type 2 diabetes?”* was followed by five different statements. The first four statements were well known, with correct response rates ranging from 90.0% to 98.0%. It is worth noting that, the fifth statement related to hereditary susceptibility *“The person’s blood sugar is slightly elevated, but they do not yet have diabetes”* (not correct) was answered correctly by only about one‐third of participants (33.3%; 32.7%) (Table 6).

The statement connected to the hereditary susceptibility *“It is possible to prevent the onset of the type 2 diabetes by means of appropriate lifestyle*” (correct) was one of the most correctly answered items (96.7%; 98.0%). However, the second question *“What does it mean if a person has a low hereditary susceptibility to type 2 diabetes?”* followed by one statement: *“Poor lifestyle choices cannot increase their risk of developing the disease”* (not correct) received fewer correct responses (86.7%; 81.6%) (Table 6).

#### 3.4.3. Section C

There was no statistically significant difference in Section C, between baseline and postsurvey time points (*p* = 0.964), with a median of 4.0 (*Q*_1_ 4.0, *Q*_3_ 5.0, *n* = 49) at baseline and 4.0 (*Q*_1_ 4.0, *Q*_3_ 5.0, *n* = 49) at the postsurvey (Table 3). There was no statistically significant gender difference (*p* = 0.882; *p* = 0.165), and Section C and age were not correlated (*p* = 0.192; *p* = 0.327) at either time point. Similarly, there was no significant difference in education levels (*p* = 0.123; *p* = 0.247) (Table 4).

### 3.5. Relationship Between Health Literacy and Genomic Literacy

There was a strong positive correlation between the health literacy scales EHIL and HLS‐EU‐Q16 index at baseline and at the postsurvey time points (*r* = 0.61, *p* < 0.001; *r* = 0.64, *p* < 0.001). Confidence showed the strongest correlation among the EHIL factors with the HLS‐EU‐Q16 index and indicated a strong positive relationship (*r* = 0.60, *p* < 0.001; *r* = 0.63, *p* < 0.001). Within genomic literacy, Sections A and C showed a strong positive correlation only at the postsurvey time point (*r* = 0.50, *p* < 0.001) (Table 7; Figure 1). No association was observed between the HLS‐EU‐Q16 index and genomic literacy at baseline (Section A *p* = 0.172, Section C *p* = 0.578) or in postsurveys (Section A *p* = 0.426, Section C *p* = 0.848). Also, at baseline, the EHIL did not correlate with genomic literacy (Section A *p* = 0.236, Section C *p* = 0.527), but at the postsurvey time point, there was a moderate positive correlation with Section A (*r* = 0.40, *p* = 0.005), and motivation (*r* = 0.35, *p* = 0.013) and confidence (*r* = 0.35, *p* = 0.013) also showed a moderate positive correlation with Section A. Genomic literacy, Section C, which focused on numeracy, was only moderately correlated with motivation at the postsurvey time point (*r* = 0.34, *p* = 0.016) (Table 7).

**TABLE 7:** Relationship between health literacy and genomic literacy, Sections A and C, in baseline and postsurvey time points.

Instruments		HLS‐EU‐Q16 index	Genomic literacy, Section A	Genomic literacy, Section C
EHIL	Baseline	*r* = 0.61, **p** ** < 0.001** ^a^	*r* = 0.16, *p* = 0.236^b^	*r* = 0.08, *p* = 0.527^b^
	Postsurvey	*r* = 0.64, **p** ** < 0.001** ^a^	*r* = 0.40, **p** ** = 0.005** ^b^	*r* = 0.22, *p* = 0.129^b^

Motivation	Baseline	*r* = 0.34, **p** ** = 0.014** ^b^	*r* = 0.11, *p* = 0.411^b^	*r* = 0.13, *p* = 0.339^b^
	Postsurvey	*r* = 0.38, **p** ** = 0.011** ^b^	*r* = 0.35, **p** ** = 0.013** ^b^	*r* = 0.34, **p** ** = 0.016** ^b^

Confidence	Baseline	*r* = 0.60, **p** ** < 0.001** ^b^	*r* = 0.18, *p* = 0.163^b^	*r* = 0.108, *p* = 0.413^b^
	Postsurvey	*r* = 0.63, **p** ** < 0.001** ^b^	*r* = 0.35, **p** ** = 0.013** ^b^	*r* = 0.16, *p* = 0.279^b^

Evaluation	Baseline	*r* = 0.36, **p** ** = 0.008** ^b^	*r* = 0.08, *p* = 0.535^b^	*r* = −0.11, *p* = 0.406^b^
	Postsurvey	*r* = 0.09, *p* = 0.575^b^	*r* = 0.00 *p* = 0.979^b^	*r* = −0.10, *p* = 0.495^b^

HLS‐EU‐Q16 index	Baseline		*r* = 0.19, *p* = 0.172^b^	*r* = 0.08, *p* = 0.578^b^
	Postsurvey		*r* = 0.12, *p* = 0.426^b^	*r* = 0.03, *p* = 0.848^b^

Genomic literacy, Section A	Baseline			*r* = 0.21, *p* = 0.107^b^
	Postsurvey			*r* = 0.50, **p** ** < 0.001** ^b^

*Note:* Statistically significant marked in bold.

^a^Pearson’s correlation.

^b^Spearman’s correlation.

**FIGURE 1:**
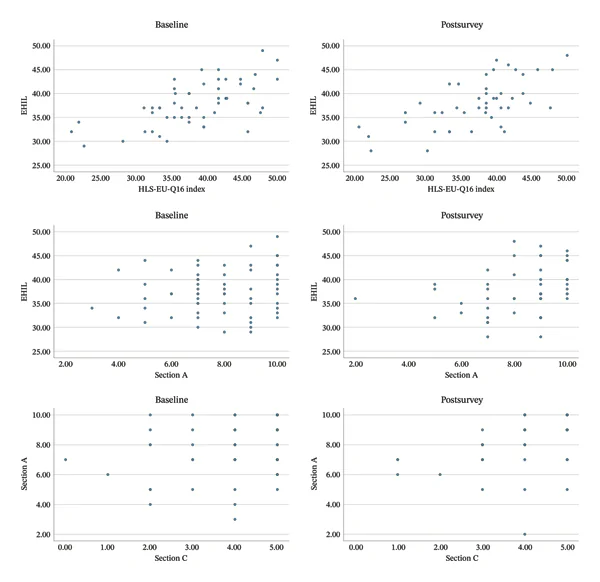
Scatterplots between EHIL and the HLS‐EU‐Q16 index, EHIL and Section A, and Sections A and C at baseline and postsurvey.

## 4. Discussion

The aim of the study was to assess participants’ health and genomic literacy. No significant change was observed in health literacy (EHIL and HLS‐EU‐Q16), suggesting that health literacy may be a relatively stable health determinant. Overall, participants demonstrated sufficient health literacy, with the HLS‐EU‐Q16 index being 38.5 (SD 6.8), which was relatively high compared with previous European studies [34, 41]. Similarly, the mean EHIL score was 37.5 (SD 4.7), which was comparable to that reported in a previous Finnish study, in which adults at increased risk for metabolic syndrome had a mean score of 36.57 (SD 4.6) [31].

Social determinants such as education and age were associated with health literacy. Participants with a higher education level scored higher on HLS‐EU‐Q16, EHIL, and confidence components. Although participants generally demonstrated sufficient health literacy, particular attention should be paid to those with the lowest educational attainment, whose mean HLS‐EU‐Q16 scores fell within the problematic range (> 25–33) [34]. Age was associated with EHIL and confidence, with health literacy declining as age increased. Although these associations were not statistically significant at every time point, they were consistent with previous research [2], and a larger sample size might have detected similar associations more consistently.

In this study, women scored significantly higher than men on EHIL and HLS‐EU‐Q16, and women’s motivation approached statistical significance at the postsurvey time point. Motivation was also of particular interest, as it correlated with all other measures at the postsurvey point (HLS‐EU‐Q16, and genomic literacy, Sections A and C). This finding may indicate that motivation reflects a greater willingness to learn and adopt new knowledge, which became more evident after intervention. However, motivation may also be influenced by factors such as risk perception and may therefore not be explained solely by high health literacy [3].

The Cronbach alpha values for EHIL were 0.67 and 0.70, which were in line with an earlier study (0.70; [32]). For HLS‐EU‐Q16, the Cronbach alpha was 0.88, which is comparatively good since prior research has reported values ranging from 0.51 to 0.91 [29]. EHIL and HLS‐EU‐Q16 expectedly correlated with each other, as both measure self‐reported health literacy.

A challenge in genomic literacy research has been that measurement has often focused on conceptual knowledge rather than the ability to apply, interpret, and use information [15]. In the present study, Section A primarily assessed understanding of genetic concepts, whereas Section B focused on the application and use of knowledge. In addition, Section C addressed genomic numeracy and the ability to interpret results. However, by including Section A, the results could be compared with previous Finnish research in which a shortened version of the instrument was also used [36], and the findings were consistent with those earlier results, supporting the reliability of this pilot study despite its small sample size. Compared with Jallinoja and Aro [35], the number of correct answers in present study were higher, which is understandable, as familiarity with genetic terms increases over time [19]. Although understanding genetic concepts may reflect general knowledge of genetics, understanding hereditary susceptibility may better capture the practical application of that knowledge, an area in which participants indeed performed better during this study.

There was an improvement in genomic literacy, Section A, during intervention. However, the clinical significance of the result should be considered, despite its statistical significance, as the increase was only one point. A similar trend was observed with age as previously reported [21, 22]: Younger participants had higher scores, and with education [3, 18, 19], those with higher education levels scored better in genomic literacy. However, at the postsurvey time point, there was no significant difference with education anymore. This gives the impression that the absorption of genomic literacy is not dependent on education level if the information is simply accessible. However, a potential ceiling effect should be considered; although scores increased among participants with a lower level of education, scores among those with the highest level of education could not increase further, as they were already at the upper end of the scale.

From Section B, the most difficult question was *“What does it mean if a person has a hereditary susceptibility to type 2 diabetes”* following the item *“The person’s blood sugar is slightly elevated, but they do not yet have diabetes”* (not correct). Only one‐third of participants answered correctly (33.3%; 32.7%), and the question format may also have contributed to the difficulty. Nevertheless, elevated blood glucose, prediabetes, and the role of hereditary susceptibility require clearer explanation during health counseling and risk communication.

Hereditary susceptibility following the statement that Type 2 diabetes can be prevented through appropriate lifestyle choices was well understood by the participants (96.7%). However, when low hereditary susceptibility was followed by the statement *“Poor lifestyle choices cannot increase their risk of developing the disease”* (incorrect), it was not as clear (86.7%). In health counseling, it is important to emphasize that maintaining a healthy lifestyle remains essential, even when hereditary risk is low.

There was no change in genomic literacy, Section C, over the intervention, and neither education, age, nor gender was associated with Section C. The most difficult item to understand was that 7% means seven out of 100 people (63.3% correct answers). Items related to the possibility of increasing or decreasing the 7% risk through lifestyle choices were clearer (80.0%–93.3%). This is indeed the most important aspect when interpreting the PRS result: Lifestyle choices influence overall risk.

Genomic literacy, Sections A and C, were also positively correlated with each other at the postsurvey time point. Also, as previously noted, Section C and motivation showed a positive correlation following the intervention. Lea et al. [13] highlighted that numeracy requires deeper information processing, and motivation is associated with attending to and seeking this type of information.

The aim of the study was also to assess the relationship between health literacy and genomic literacy. The HLS‐EU‐Q16 index did not correlate with genomic literacy, Sections A and C, but at the postsurvey, a positive moderate correlation was seen between EHIL, motivation, and confidence, with genomic literacy, Section A. In the future, it would be interesting to examine whether this association strengthens as more genomic information becomes available.

### 4.1. Strength and Limitations of the Work

A key strength of the study was the simultaneous use of two different health literacy and genomic literacy measures, together with the longitudinal design, which increases the robustness of the findings. Nevertheless, the small sample size limits the statistical power and generalizability of the findings, and even statistically significant findings should be interpreted with caution given the exploratory nature of this pilot study. Multiple testing may also increase the risk of Type I errors. Corrections for multiple comparisons were applied in pairwise (post hoc) comparisons following one‐way ANOVA and Kruskal–Wallis tests across educational levels, but not in all analyses.

Cronbach’s alpha should be interpreted with caution. The lower Cronbach’s alpha values of the EHIL instrument and its factors may be due to the multidimensionality of the instrument [33, 42] or due to the limited number of items [42]. The EHIL instrument included 10 items, and its factors consisted of only 2 to 4 items. Furthermore, as Section C was preliminary in this study, it requires further psychometric validation in a larger independent cohort.

The time between baseline surveys and postsurveys varied depending on the timing of recruitment, but the time from PRS result to postsurveys were common for all participants. Individual factors affecting the outcomes may have occurred between the baseline and postsurvey assessments but could not be measured or identified. Among other things, website use could not be assessed. Participants were also entitled to receive standard healthcare according to their individual situation.

The majority of participants were female, and the proportion of female increased further in the postsurvey. For example, participation motivation may have been higher among women, which may have introduced bias into the results. Future studies should aim for a more balanced gender distribution. Without more detailed information on the reasons behind participants’ work situations, the effect of this factor cannot be reliably assessed. Despite the slightly lower level of education, health literacy in this sample did not appear to be lower compared to previous studies. The inclusion criterion of an elevated risk of Type 2 diabetes may also have influenced participation and motivation, as awareness of risk and perceived threat is known to influence health behavior [43, 44].

### 4.2. Recommendations for Further Research

Health and genomic literacy and the factors influencing them should be studied in a larger sample to confirm the results. Further research is also needed to examine the impact of different genomic risk levels on participants to enable more personalized counseling. In this study, postsurveys were conducted 2 weeks after PRS disclosure; therefore, the long‐term effects on literacy require further research and longer follow‐up.

## 5. Conclusion

From a feasibility perspective, it was noteworthy that even limited health counseling and risk information may increase genomic literacy. We recommend that PRS results be disclosed in person and combined with lifestyle counseling. Personal counseling allows the results to be explained according to the participant’s health literacy and level of understanding.

## Author Contributions

Elisa Airikkala: formal analysis, writing–original draft, and writing–review and editing.

Marja Kaunonen: writing–review and editing, supervision.

Anna‐Maija Koivisto: formal analysis and writing–review and editing.

Elina Pimiä: data curation and writing–review and editing.

Arja Halkoaho: writing–review and editing and supervision.

## Funding

The survey data used in this study were collected as part of the DIGIDIA project, which was funded by the European Social Fund (ESF). The subsequent data analyses and the writing of this article were conducted independently without external funding support.

## Conflicts of Interest

The authors declare no conflicts of interest.

## Supporting Information

Additional supporting information can be found online in the Supporting Information section.

## Supporting information


**Supporting Information** STROBE Statement—Checklist of items that should be included in reports of cross‐sectional studies.

## Data Availability

The data that support the findings of this study are not publicly available due to their containing information that could compromise the privacy of research participants but are available with restrictions from the corresponding author.
